# Crystal structure of [{[Ni(C_10_H_24_N_4_)][Ni(CN)_4_]}·2H_2_O]_*n*_, a one-dimensional coordination polymer formed from the [Ni(cyclam)]^2+^ cation and the [Ni(CN)_4_]^2–^ anion

**DOI:** 10.1107/S2056989021010902

**Published:** 2021-10-21

**Authors:** Liudmyla V. Tsymbal, Irina L. Andriichuk, Sergiu Shova, Yaroslaw D. Lampeka

**Affiliations:** a L. V. Pisarzhevskii Institute of Physical Chemistry of the National Academy of Sciences of Ukraine, Prospekt Nauki 31, Kyiv, 03028, Ukraine; b "Petru Poni" Institute of Macromolecular Chemistry, Department of Inorganic Polymers, Aleea Grigore Ghika Voda41A, RO-700487 Iasi, Romania

**Keywords:** crystal structure, coordination polymer, cyclam, nickel, hydrogen bonds

## Abstract

The asymmetric unit of the title compound, *catena*-poly[[[(1,4,8,11-tetra­aza­cyclo­tetra­decane-κ^4^ *N* ^1^,*N* ^4^,*N* ^8^,*N* ^11^)nickel(II)]-μ-cyanido-κ^2^ *N*:*C*-[bis­(cyanido-κ*C*)nickel(II)]-μ-cyanido-κ^2^ *C*:*N*] dihydrate], {[Ni_2_(CN)_4_(C_10_H_24_N_4_)]·2H_2_O]_*n*_ or [{[Ni(C_10_H_24_N_4_)][Ni(CN)_4_]}·2H_2_O]_*n*_, consists of a pair of crystallographically non-equivalent macrocyclic cations and anions. The nickel(II) ions (all with site symmetry  ) are coordinated by the four secondary N atoms of the macrocyclic ligands, which adopt the most energetically stable *trans*-III conformation, and the mutually *trans* N atoms of the tetra­cyano­nickelate anion in a slightly tetra­gonally distorted NiN_6_ octa­hedral coordination geometry. The [Ni(CN)_4_)]^2–^ anion exhibits a bridging function, resulting in the formation of parallel polymeric chains running along the [1  0] direction. The water mol­ecules of crystallization play a pivotal role in the three-dimensional supra­molecular organization of the crystal. Acting as acceptors, they form N—H⋯O_w_ (w = water) hydrogen bonds with the secondary amino groups of the macrocycles, forming layers oriented parallel to the (001) plane. At the same time, as donors, they inter­act with the non-coordinated cyano groups of the anion *via* O_w_—H⋯N_c_ (c = cyanide) hydrogen bonds, giving two-dimensional layers oriented parallel to the (100) plane and thus generating a three-dimensional network.

## Abstract 2

The title coordination polymer consists of parallel linear chains built up of macrocyclic cations possessing a slightly tetra­gonally distorted NiN_6_ octa­hedral coordination geometry formed by four N atoms of the aza­macrocyclic ligand in the equatorial plane and two *trans* N atoms of the cyanide groups of the bridging tetra­cyano­nickelate anion in the axial positions. In the crystal, two independent [1 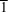 0] polymeric chains are cross-linked by N—H⋯O_w_ (w = water) and O_w_—H⋯N_c_ (c = cyanide) hydrogen bonds into a three-dimensional network.

## Chemical context

Transition-metal complexes of tetra­aza­macrocyclic ligands, in particular of 1,4,8,11-tetra­aza­cyclo­tetra­decane (cyclam, *L*), have been intensively studied for decades. This is explained by their unique properties, in particular, exceptionally high thermodynamic stability, kinetic inertness and the ability to stabilize uncommon oxidation states of coordinated metals (Melson, 1979 ▸; Yatsimirskii & Lampeka, 1985 ▸). Because of their conformational rigidity during chemical transformation (preservation of two vacant or labile *trans* axial positions in the coordination sphere of the metal ion), these complexes are also promising secondary building units for the construction of metal–organic frameworks (MOFs) (Lampeka & Tsymbal, 2004 ▸; Suh & Moon, 2007 ▸; Suh *et al.*, 2012 ▸; Stackhouse & Ma, 2018 ▸), which possess great potential for applications in different areas including gas storage, separation, catalysis, sensing, *etc* (MacGillivray & Lukehart, 2014 ▸; Kaskel, 2016 ▸).

Cyano­metallate anions refer to a type of bridging ligands for the creation of MOFs of different topologies possessing promising magnetic and electronic properties (Ohkoshi *et al.*, 2019 ▸). Among such linkers, the tetra­cyano­nickelate(II) dianion has attracted less attention compared to hexa- and octa­cyano­metallates and only one work describing the structure of the coordination polymer formed by the metal(cyclam) complex and this anion, *i.e*., {Cu(*L*)[Ni(CN)_4_]}_*n*_, has been published to date (Černák *et al.*, 2010 ▸). Inter­estingly, despite the diamagnetic nature of the bridging fragment, this complex displays a weak anti­ferromagnetic exchange coupling between the paramagnetic copper(II) centres. 
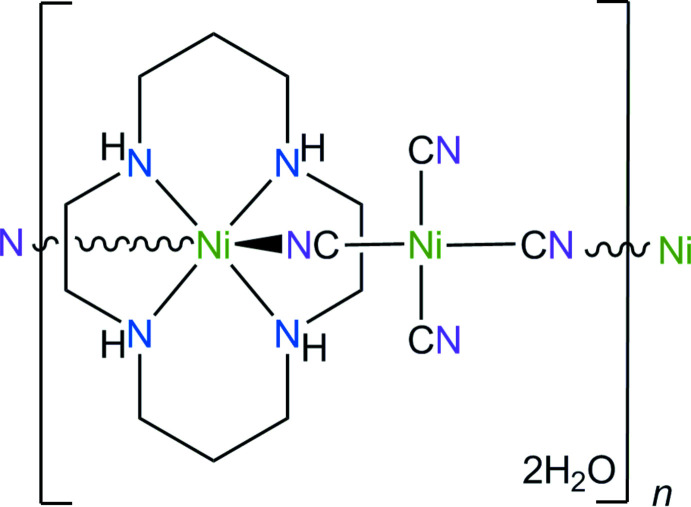


We report herein the synthesis and crystal structure of the coordination polymer built up of the nickel(II) complex of *L* and the tetra­cyano­nickelate(II) dianion, namely, *catena*-[bis­(μ_2_-cyano-κ^2^C,N)-di­cyano-(1,4,8,11-tetra­aza­cyclo­tetra­dec­ane-κ^4^N^1^,N^4^,N^8^,N^11^)-dinickel(II) dihydrate], [{[Ni(*L*)][Ni(CN)_4_]}·2H_2_O]_*n*_, (**I**).

## Structural commentary

**Figure 1:**
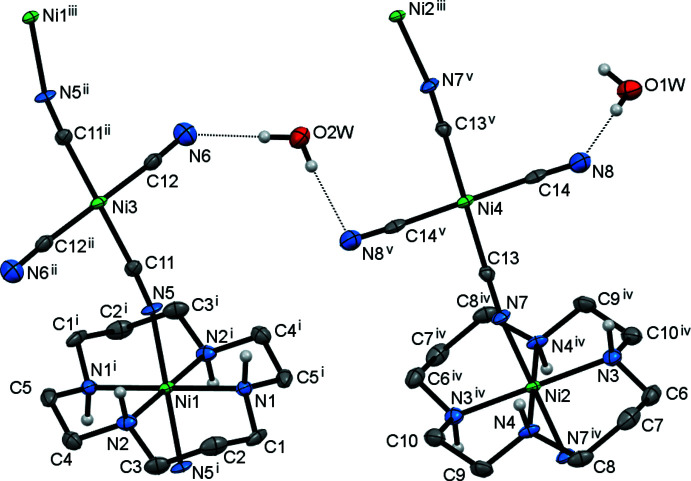
The extended asymmetric unit in **I** showing the coordination environment of the Ni atoms and the atom-labelling scheme (displacement ellipsoids are drawn at the 40% probability level). C-bound H atoms are omitted for clarity. Dotted lines represent hydrogen-bonding inter­actions. Symmetry codes: (i) −*x* + 1, −*y* + 1, −*z*; (ii) −*x*, −*y* + 2, −*z*; (iii) *x* − 1, *y* + 1, *z*; (iv) −*x* + 1, −*y*, −*z* + 1; (v) −*x*, −*y* + 1, −*z* + 1.

The mol­ecular structure of **I** is shown in Fig. 1 ▸. It represents a one-dimensional coordination polymer built up from two crystallographically independent centrosymmetric tetra­gonal macrocyclic [Ni(*L*)]^2+^ cations and tetra­cyano­nickelate anions [Ni(CN)_4_]^2–^. The coordination of the *trans* cyanide groups of the anions in the axial positions of the coordination sphere of the metal ions in cations results in the formation of two structurally non-equivalent parallel polymeric chains (Ni1/Ni3 and Ni2/Ni4) running along the [1 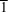 0] direction.

The location of the metal ions on inversion centres enforces strict planarity of the Ni(N_4_) and Ni(C_4_) coordination moieties. The macrocyclic ligand in the complex cations adopts the most common and energetically favorable *trans*-III (*R*,*R*,*S*,*S*) conformation (Bosnich *et al.*, 1965 ▸) with almost equal Ni—N bond lengths (Table 1 ▸). The five-membered chelate rings are present in *gauche* (bite angles *ca* 85.5°) and the six-membered in *chair* (bite angles *ca* 94.5°) conformations (Table 1 ▸). The geometric parameters observed are characteristic of high-spin *d* ^8^ nickel(II) complexes with macrocyclic 14-membered tetra­amine ligands (Lampeka & Tsymbal, 2004 ▸; Tsymbal *et al.*, 2021 ▸). The axial Ni—N(CN) bond lengths are somewhat longer than the Ni—N(amine) ones, resulting in a slight tetra­gonal distortion of the *trans*-NiN_4_N_2_ coordination polyhedron.

The Ni—C—N angles in the anion deviate only slightly (less than 4°) from linearity. In **I**, each tetra­cyano­nickelate unit uses two *trans* cyanide groups for coordination to two macrocyclic moieties in a bent fashion [Ni—N—C = 166.1 (4)°], giving rise to a linear polymeric chain, whereas the two remaining *trans* CN^−^ groups are monodentate. The adjacent Ni⋯Ni distance in the chain is 5.0558 (5) Å, and the shortest inter­chain Ni⋯Ni distance is 6.6159 (5) Å.

## Supra­molecular features

**Figure 2:**
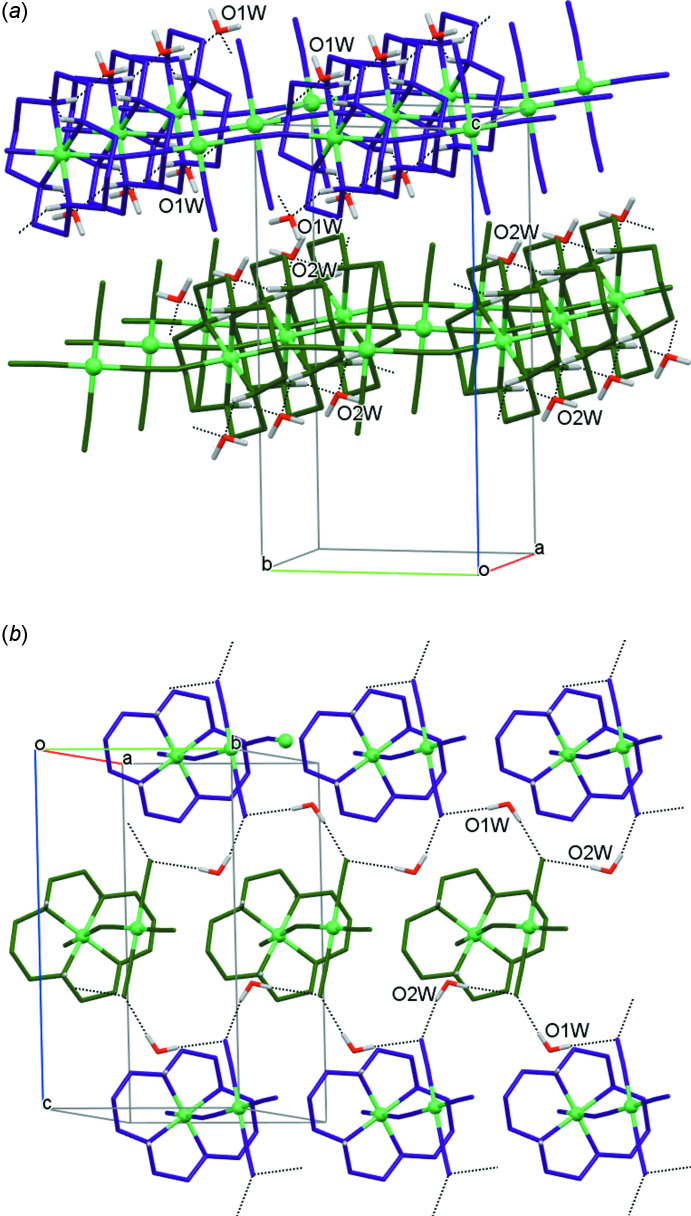
View of the sheets of polymeric chains formed due to the hydrogen-bond acceptor (*a*) and donor (*b*) properties of the water mol­ecules of crystallization. The macrocyclic ligands in the crystallographically non-equivalent nickel ions are shown in violet (Ni1/Ni3) and green (Ni2/Ni4) colors.

The crystals of **I** are composed of linear polymeric chains of [Ni(*L*)]^2+^ cations bridged by the [Ni(CN)_4_]^2−^ anions, which propagate along the [1 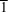 0] direction. There are no direct contacts between the chains and the water mol­ecules of crystallization play a key role in assembling them into a three-dimensional supra­molecular network. In particular, serving as the acceptor for N—H⋯O hydrogen bonds arising from the secondary amino groups of different macrocyclic ligands in the crystallographically equivalent chains (O1*W* for Ni1/Ni3, O2*W* for Ni2/Ni4), the water mol­ecules link them in two-dimensional layers oriented parallel to the (001) plane (Table 2 ▸, Fig. 2 ▸ *a*). At the same time, acting as the donors in O—H⋯N hydrogen-bonding inter­actions with the nitro­gen atoms of the non-coordinating cyanide groups of the anions belonging to crystallographically non-equivalent polymeric chains, they form two-dimensional layers oriented parallel to the (100) plane (Table 2 ▸, Fig. 2 ▸ *b*) thus realizing a three-dimensional system of hydrogen bonds in the crystal.

## Database survey

A search of the Cambridge Structural Database (CSD, version 5.42, last update February 2021; Groom *et al.*, 2016 ▸) indicated that several one-dimensional coordination polymers formed by di­aza­cyclam nickel(II) cations (di­aza­cyclam = 1,3,5,8,10,12-hexa­aza­cyclo­tetra­deca­ne) and the tetra­cyano­nickelate anion have been characterized structurally. They include compounds with monomacrocylic (refcode MIMJIB; Kou *et al.*, 2002 ▸) and macrotricyclic [NADVOE (Zhou *et al.*, 2004 ▸), YUBHEK, YUBHIO and YUBHOU (Jiang *et al.*, 2015 ▸)] tetra­dentate ligands. The structures of the polymeric chains in these compounds are very similar. In particular, because of comparable Ni—N(CN) bond lengths and Ni—N—C bond angles, the inter­chain Ni⋯Ni distances fall in the range 5.07–5.15 Å and are slightly longer than that observed in **I**. Surprisingly, a similar value for this parameter (5.056 Å) is also observed in the complex of the [Cu(*L*)]^2+^ cation with [Ni(CN)_4_]^2−^ (XABGEO; Černák *et al.*, 2010 ▸), despite the substanti­ally longer Cu—N(CN) distance (2.532 Å). This feature is explained by the considerable bending of the Cu—N—C (133.0°) angle as compared the nickel analogues.

## Synthesis and crystallization

All reagents and solvents used in this work were analytical grade and were used without further purification. The macrocyclic nickel(II) complex Ni(*L*)(ClO_4_)_2_ was prepared according to procedures described previously (Barefield *et al.*, 1976 ▸).

**[{[Ni(** ***L*** **)][Ni(CN)_4_]}·2H_2_O]** ***_n_*** **, (I):** A solution of 121 mg (0.50 mmol) of K_2_[Ni(CN)_4_] in 15 ml of water was added under stirring to a solution of 290 mg (0.50 mmol) Ni(*L*)(ClO_4_)_2_ in 10 ml of di­methyl­formamide. Filtration and slow evaporation of the resulting solution gave after several days a light-yellow crystalline precipitate, which was washed with DMF, methanol and dried in air. Yield 160 mg (35%). Analysis calculated for C_14_H_28_N_8_Ni_2_O_2_: C, 36.72; H, 6.16; N, 24.47%. Found: C, 36.62; H, 6.26; N, 24.19%. Single crystals suitable for X-ray diffraction analysis were selected from the sample resulting from the synthesis. **Safety note**: perchlorate salts of metal complexes are potentially explosive and should be handled with care.

## Refinement

Crystal data, data collection and structure refinement details are summarized in Table 3 ▸. All H atoms in **I** were placed in geometrically idealized positions and constrained to ride on their parent atoms, with C—H = 0.97 Å, N—H = 0.98 Å and water O—H = 0.85 Å, with *U*_iso_(H) values of 1.2 or 1.5U_eq_ of the parent atoms.

## Supplementary Material

Crystal structure: contains datablock(s) I. DOI: 10.1107/S2056989021010902/hb7992sup1.cif

Structure factors: contains datablock(s) I. DOI: 10.1107/S2056989021010902/hb7992Isup2.hkl

CCDC reference: 2114932

Additional supporting information:  crystallographic information; 3D view; checkCIF report

## Figures and Tables

**Table 1:** Selected bond lengths (Å)

Ni1—N5	2.100 (4)	Ni2—N4	2.079 (4)
Ni1—N2	2.070 (4)	Ni3—C11	1.874 (6)
Ni1—N1	2.082 (4)	Ni3—C12	1.857 (6)
Ni2—N3	2.069 (4)	Ni4—C13	1.866 (6)
Ni2—N7	2.095 (4)	Ni4—C14	1.863 (6)

**Table 2:** Hydrogen-bond geometry (Å, °)

*D*—H⋯*A*	*D*—H	H⋯*A*	*D*⋯*A*	*D*—H⋯*A*
N1—H1⋯O1*W* ^i^	0.98	2.28	3.115 (6)	143
N2—H2⋯O1*W* ^ii^	0.98	2.10	3.020 (6)	156
N3—H3⋯O2*W* ^i^	0.98	2.15	3.083 (7)	159
N4—H4⋯O2*W* ^iii^	0.98	2.26	3.080 (6)	140
O1*W*—H1*WA*⋯N8	0.85	2.03	2.872 (7)	173
O1*W*—H1*WB*⋯N6^iv^	0.85	2.27	3.112 (7)	171
O2*W*—H2*WA*⋯N6	0.85	2.03	2.853 (6)	164
O2*W*—H2*WB*⋯N8^i^	0.85	2.30	3.149 (7)	175

Symmetry codes: (i) -x, -y+1, -z+1; (ii) x, y, z-1; (iii) x, y-1, z; (iv) -x, -y+2, -z+1.

**Table 3:** Experimental details

Crystal data	
Chemical formula	[Ni_2_(CN)_4_(C_10_H_24_N_4_)]·2H_2_O
*M*_r_	457.86
Crystal system, space group	Triclinic, *P*\overline{1}
Temperature (K)	180
*a*, *b*, *c* (Å)	7.7325 (6), 8.8809 (7), 15.7780 (12)
α, β, γ (°)	88.673 (6), 85.682 (7), 74.623 (7)
*V* (Å^3^)	1041.74 (15)
*Z*	2
Radiation type	Mo *K*α
μ (mm^−1^)	1.83
Crystal size (mm)	0.30 × 0.20 × 0.06

Data collection	
Diffractometer	Rigaku Xcalibur, Eos
Absorption correction	Multi-scan (*CrysAlis PRO*; Rigaku OD, 2018 ▸)
*T*_min_, *T*_max_	0.852, 1.000
No. of measured, independent and observed [*I* > 2σ(*I*)] reflections	7144, 3673, 2417
*R*_int_	0.036
(sin θ/λ)_max_ (Å^−1^)	0.595

Refinement	
*R*[*F* ^2^ > 2σ(*F* ^2^)], *wR*(*F* ^2^), *S*	0.059, 0.159, 1.07
No. of reflections	3673
No. of parameters	247
H-atom treatment	H-atom parameters constrained
Δρ_max_, Δρ_min_ (e Å^−3^)	1.31, −0.42

Computer programs: *CrysAlis PRO* (Rigaku OD, 2018 ▸), *SHELXT2018/2* (Sheldrick, 2015*a* ▸), *SHELXL2018/3* (Sheldrick, 2015*b* ▸), *Mercury* (Macrae *et al.*, 2020 ▸) and *publCIF* (Westrip, 2010 ▸).

## supplementary crystallographic information

### Crystal data

**Table:**

[Ni_2_(CN)_4_(C_10_H_24_N_4_)]·2H_2_O	*Z* = 2
*M**_r_* = 457.86	*F*(000) = 480
Triclinic, *P*1	*D*_x_ = 1.460 Mg m^−^^3^
*a* = 7.7325 (6) Å	Mo *K*α radiation, λ = 0.71073 Å
*b* = 8.8809 (7) Å	Cell parameters from 2303 reflections
*c* = 15.7780 (12) Å	θ = 2.7–28.8°
α = 88.673 (6)°	µ = 1.83 mm^−^^1^
β = 85.682 (7)°	*T* = 180 K
γ = 74.623 (7)°	Block, colourless
*V* = 1041.74 (15) Å^3^	0.30 × 0.20 × 0.06 mm

### Data collection

**Table:**

Rigaku Xcalibur, Eos diffractometer	3673 independent reflections
Radiation source: fine-focus sealed X-ray tube, Enhance (Mo) X-ray Source	2417 reflections with *I* > 2σ(*I*)
Graphite monochromator	*R*_int_ = 0.036
Detector resolution: 8.0797 pixels mm^-1^	θ_max_ = 25.0°, θ_min_ = 2.4°
ω scans	*h* = −9→9
Absorption correction: multi-scan (CrysAlisPro; Rigaku OD, 2018)	*k* = −9→10
*T*_min_ = 0.852, *T*_max_ = 1.000	*l* = −17→18
7144 measured reflections	

### Refinement

**Table:**

Refinement on *F*^2^	Primary atom site location: dual
Least-squares matrix: full	Hydrogen site location: mixed
*R*[*F*^2^ > 2σ(*F*^2^)] = 0.059	H-atom parameters constrained
*wR*(*F*^2^) = 0.159	*w* = 1/[σ^2^(*F*_o_^2^) + (0.064*P*)^2^ + 1.1599*P*] where *P* = (*F*_o_^2^ + 2*F*_c_^2^)/3
*S* = 1.07	(Δ/σ)_max_ < 0.001
3673 reflections	Δρ_max_ = 1.31 e Å^−^^3^
247 parameters	Δρ_min_ = −0.42 e Å^−^^3^
0 restraints	

### Special details

**Table:**

Geometry. All esds (except the esd in the dihedral angle between two l.s. planes) are estimated using the full covariance matrix. The cell esds are taken into account individually in the estimation of esds in distances, angles and torsion angles; correlations between esds in cell parameters are only used when they are defined by crystal symmetry. An approximate (isotropic) treatment of cell esds is used for estimating esds involving l.s. planes.

### Fractional atomic coordinates and isotropic or equivalent isotropic displacement parameters (Å^2^)

**Table:**

	*x*	*y*	*z*	*U*_iso_*/*U*_eq_	
Ni4	0.000000	0.500000	0.500000	0.0201 (3)	
Ni3	0.000000	1.000000	0.000000	0.0195 (3)	
Ni1	0.500000	0.500000	0.000000	0.0172 (3)	
Ni2	0.500000	0.000000	0.500000	0.0177 (3)	
C13	0.1682 (7)	0.3061 (6)	0.4882 (3)	0.0190 (11)	
N5	0.2721 (5)	0.6907 (5)	0.0165 (3)	0.0232 (10)	
C8	0.3761 (7)	−0.2867 (6)	0.4659 (4)	0.0312 (14)	
H8A	0.496131	−0.353457	0.472014	0.037*	
H8B	0.316556	−0.338924	0.428528	0.037*	
N8	−0.0359 (7)	0.4416 (6)	0.6889 (3)	0.0424 (14)	
N3	0.3772 (6)	−0.0524 (5)	0.6138 (3)	0.0259 (11)	
H3	0.254063	0.015213	0.617490	0.031*	
N6	−0.0089 (7)	1.0667 (7)	0.1866 (3)	0.0459 (14)	
N2	0.4008 (6)	0.4404 (5)	−0.1091 (3)	0.0247 (11)	
H2	0.278118	0.507374	−0.111458	0.030*	
C11	0.1664 (7)	0.8059 (7)	0.0125 (3)	0.0205 (12)	
O2W	0.0288 (6)	0.9077 (5)	0.3461 (2)	0.0374 (10)	
H2WA	0.036923	0.959680	0.300850	0.056*	
H2WB	0.032480	0.814393	0.333553	0.056*	
C14	−0.0241 (7)	0.4667 (6)	0.6168 (4)	0.0273 (13)	
C10	0.5288 (8)	0.0021 (7)	0.3194 (4)	0.0322 (14)	
H10A	0.601399	−0.007329	0.265908	0.039*	
H10B	0.415596	0.079153	0.312246	0.039*	
N1	0.3727 (5)	0.3666 (5)	0.0802 (3)	0.0224 (10)	
H1	0.247298	0.427127	0.090795	0.027*	
C3	0.3903 (7)	0.2784 (7)	−0.1143 (4)	0.0336 (14)	
H3A	0.510949	0.209177	−0.117835	0.040*	
H3B	0.334252	0.265566	−0.165612	0.040*	
N7	0.2769 (5)	0.1898 (5)	0.4838 (3)	0.0231 (10)	
C4	0.5107 (7)	0.4876 (7)	−0.1807 (3)	0.0289 (14)	
H4A	0.448916	0.495059	−0.232483	0.035*	
H4B	0.625088	0.409578	−0.189027	0.035*	
C12	−0.0032 (7)	1.0368 (6)	0.1155 (4)	0.0273 (13)	
O1W	0.0212 (6)	0.5830 (5)	0.8426 (3)	0.0383 (11)	
H1WA	−0.004667	0.541681	0.798936	0.057*	
H1WB	0.027685	0.675211	0.830710	0.057*	
C1	0.3698 (7)	0.2153 (6)	0.0457 (4)	0.0300 (14)	
H1A	0.303586	0.163745	0.086184	0.036*	
H1B	0.491913	0.149679	0.037738	0.036*	
N4	0.3896 (5)	−0.1378 (5)	0.4269 (3)	0.0225 (10)	
H4	0.267023	−0.077143	0.417091	0.027*	
C2	0.2837 (7)	0.2331 (7)	−0.0382 (4)	0.0373 (15)	
H2A	0.169263	0.311348	−0.031187	0.045*	
H2B	0.258498	0.135002	−0.050764	0.045*	
C6	0.3638 (7)	−0.2135 (7)	0.6248 (4)	0.0336 (15)	
H6A	0.295970	−0.222107	0.678108	0.040*	
H6B	0.483387	−0.283061	0.627483	0.040*	
C5	0.5424 (7)	0.6439 (7)	−0.1614 (3)	0.0295 (14)	
H5A	0.619798	0.672475	−0.206694	0.035*	
H5B	0.428831	0.723764	−0.157782	0.035*	
C9	0.4941 (7)	−0.1553 (7)	0.3439 (3)	0.0323 (14)	
H9A	0.427445	−0.187587	0.301267	0.039*	
H9B	0.607290	−0.234112	0.347689	0.039*	
C7	0.2724 (7)	−0.2638 (7)	0.5524 (4)	0.0364 (16)	
H7A	0.244558	−0.361196	0.568747	0.044*	
H7B	0.159067	−0.186226	0.546429	0.044*	

### Atomic displacement parameters (Å^2^)

**Table:**

	*U*^11^	*U*^22^	*U*^33^	*U*^12^	*U*^13^	*U*^23^
Ni4	0.0180 (5)	0.0115 (5)	0.0275 (6)	0.0020 (4)	−0.0016 (4)	−0.0006 (4)
Ni3	0.0170 (5)	0.0117 (5)	0.0265 (6)	0.0015 (4)	−0.0005 (4)	−0.0005 (4)
Ni1	0.0152 (5)	0.0111 (5)	0.0234 (6)	−0.0001 (4)	−0.0012 (4)	−0.0006 (4)
Ni2	0.0177 (5)	0.0099 (5)	0.0228 (6)	0.0010 (4)	−0.0005 (4)	−0.0019 (4)
C13	0.017 (3)	0.020 (3)	0.020 (3)	−0.006 (2)	0.002 (2)	−0.001 (2)
N5	0.015 (2)	0.012 (3)	0.037 (3)	0.005 (2)	−0.0038 (19)	−0.002 (2)
C8	0.024 (3)	0.020 (3)	0.051 (4)	−0.007 (3)	−0.007 (3)	−0.009 (3)
N8	0.058 (4)	0.029 (3)	0.037 (3)	−0.005 (3)	−0.004 (3)	−0.001 (3)
N3	0.022 (2)	0.025 (3)	0.029 (3)	−0.002 (2)	−0.0016 (19)	0.002 (2)
N6	0.055 (4)	0.045 (4)	0.037 (4)	−0.012 (3)	0.000 (3)	−0.002 (3)
N2	0.020 (2)	0.023 (3)	0.028 (3)	−0.001 (2)	−0.0050 (18)	−0.003 (2)
C11	0.024 (3)	0.019 (3)	0.020 (3)	−0.007 (2)	−0.006 (2)	−0.001 (2)
O2W	0.041 (2)	0.030 (3)	0.039 (3)	−0.004 (2)	−0.0061 (19)	0.005 (2)
C14	0.020 (3)	0.014 (3)	0.044 (4)	0.004 (2)	−0.005 (2)	−0.002 (3)
C10	0.035 (3)	0.029 (4)	0.029 (3)	−0.001 (3)	−0.002 (2)	−0.005 (3)
N1	0.022 (2)	0.018 (3)	0.026 (3)	−0.0045 (19)	0.0040 (18)	0.000 (2)
C3	0.032 (3)	0.025 (4)	0.046 (4)	−0.008 (3)	−0.008 (3)	−0.011 (3)
N7	0.017 (2)	0.014 (3)	0.034 (3)	0.002 (2)	−0.0014 (18)	0.003 (2)
C4	0.028 (3)	0.034 (4)	0.023 (3)	−0.006 (3)	−0.001 (2)	−0.003 (3)
C12	0.027 (3)	0.015 (3)	0.039 (4)	−0.005 (2)	0.003 (2)	0.005 (3)
O1W	0.038 (2)	0.033 (3)	0.042 (3)	−0.003 (2)	−0.0109 (19)	−0.006 (2)
C1	0.019 (3)	0.012 (3)	0.057 (4)	−0.004 (2)	0.008 (2)	0.007 (3)
N4	0.021 (2)	0.013 (2)	0.034 (3)	−0.0037 (19)	−0.0065 (18)	−0.006 (2)
C2	0.025 (3)	0.026 (4)	0.064 (4)	−0.011 (3)	−0.003 (3)	−0.010 (3)
C6	0.027 (3)	0.027 (4)	0.045 (4)	−0.006 (3)	0.003 (3)	0.010 (3)
C5	0.024 (3)	0.030 (4)	0.028 (3)	0.002 (3)	0.003 (2)	0.009 (3)
C9	0.038 (3)	0.030 (4)	0.026 (3)	−0.002 (3)	−0.004 (2)	−0.012 (3)
C7	0.018 (3)	0.027 (4)	0.064 (5)	−0.008 (3)	−0.002 (3)	0.010 (3)

### Geometric parameters (Å, º)

**Table:**

Ni1—N5	2.100 (4)	N2—C4	1.482 (7)
Ni1—N5^i^	2.100 (4)	O2W—H2WA	0.8491
Ni1—N2^i^	2.070 (4)	O2W—H2WB	0.8493
Ni1—N2	2.070 (4)	C10—H10A	0.9700
Ni1—N1^i^	2.082 (4)	C10—H10B	0.9700
Ni1—N1	2.082 (4)	C10—C9	1.528 (8)
Ni2—N3^ii^	2.069 (4)	N1—H1	0.9800
Ni2—N3	2.069 (4)	N1—C1	1.468 (7)
Ni2—N7	2.095 (4)	N1—C5^i^	1.474 (6)
Ni2—N7^ii^	2.095 (4)	C3—H3A	0.9700
Ni2—N4	2.079 (4)	C3—H3B	0.9700
Ni2—N4^ii^	2.079 (4)	C3—C2	1.512 (8)
Ni3—C11	1.874 (6)	C4—H4A	0.9700
Ni3—C11^iii^	1.874 (6)	C4—H4B	0.9700
Ni3—C12^iii^	1.857 (6)	C4—C5	1.514 (8)
Ni3—C12	1.857 (6)	O1W—H1WA	0.8499
Ni4—C13^iv^	1.866 (6)	O1W—H1WB	0.8492
Ni4—C13	1.866 (6)	C1—H1A	0.9700
Ni4—C14^iv^	1.863 (6)	C1—H1B	0.9700
Ni4—C14	1.863 (6)	C1—C2	1.511 (8)
C13—N7	1.145 (7)	N4—H4	0.9800
N5—C11	1.132 (7)	N4—C9	1.475 (7)
C8—H8A	0.9700	C2—H2A	0.9700
C8—H8B	0.9700	C2—H2B	0.9700
C8—N4	1.470 (6)	C6—H6A	0.9700
C8—C7	1.521 (8)	C6—H6B	0.9700
N8—C14	1.156 (7)	C6—C7	1.521 (8)
N3—H3	0.9800	C5—H5A	0.9700
N3—C10^ii^	1.466 (7)	C5—H5B	0.9700
N3—C6	1.468 (7)	C9—H9A	0.9700
N6—C12	1.154 (7)	C9—H9B	0.9700
N2—H2	0.9800	C7—H7A	0.9700
N2—C3	1.467 (7)	C7—H7B	0.9700
			
C13^iv^—Ni4—C13	180.0	N3^ii^—C10—C9	109.3 (4)
C14^iv^—Ni4—C13^iv^	89.7 (2)	H10A—C10—H10B	108.3
C14—Ni4—C13^iv^	90.3 (2)	C9—C10—H10A	109.8
C14^iv^—Ni4—C13	90.3 (2)	C9—C10—H10B	109.8
C14—Ni4—C13	89.7 (2)	Ni1—N1—H1	107.1
C14^iv^—Ni4—C14	180.0	C1—N1—Ni1	115.1 (3)
C11—Ni3—C11^iii^	180.0	C1—N1—H1	107.1
C12—Ni3—C11	90.1 (2)	C1—N1—C5^i^	114.3 (4)
C12^iii^—Ni3—C11^iii^	90.1 (2)	C5^i^—N1—Ni1	105.7 (3)
C12—Ni3—C11^iii^	89.9 (2)	C5^i^—N1—H1	107.1
C12^iii^—Ni3—C11	89.9 (2)	N2—C3—H3A	109.2
C12^iii^—Ni3—C12	180.0	N2—C3—H3B	109.2
N5^i^—Ni1—N5	180.0	N2—C3—C2	111.9 (5)
N2—Ni1—N5	89.31 (17)	H3A—C3—H3B	107.9
N2—Ni1—N5^i^	90.69 (17)	C2—C3—H3A	109.2
N2^i^—Ni1—N5^i^	89.31 (17)	C2—C3—H3B	109.2
N2^i^—Ni1—N5	90.69 (17)	C13—N7—Ni2	166.1 (4)
N2^i^—Ni1—N2	180.00 (11)	N2—C4—H4A	109.8
N2—Ni1—N1^i^	85.61 (17)	N2—C4—H4B	109.8
N2^i^—Ni1—N1	85.61 (17)	N2—C4—C5	109.6 (5)
N2^i^—Ni1—N1^i^	94.39 (17)	H4A—C4—H4B	108.2
N2—Ni1—N1	94.39 (17)	C5—C4—H4A	109.8
N1^i^—Ni1—N5	90.34 (17)	C5—C4—H4B	109.8
N1—Ni1—N5	89.66 (17)	N6—C12—Ni3	176.9 (5)
N1^i^—Ni1—N5^i^	89.66 (17)	H1WA—O1W—H1WB	109.4
N1—Ni1—N5^i^	90.34 (17)	N1—C1—H1A	109.2
N1^i^—Ni1—N1	180.0	N1—C1—H1B	109.2
N3^ii^—Ni2—N3	180.0	N1—C1—C2	111.9 (4)
N3^ii^—Ni2—N7^ii^	89.44 (17)	H1A—C1—H1B	107.9
N3—Ni2—N7^ii^	90.56 (17)	C2—C1—H1A	109.2
N3^ii^—Ni2—N7	90.56 (17)	C2—C1—H1B	109.2
N3—Ni2—N7	89.44 (17)	Ni2—N4—H4	107.0
N3^ii^—Ni2—N4	85.36 (17)	C8—N4—Ni2	115.6 (3)
N3—Ni2—N4^ii^	85.36 (17)	C8—N4—H4	107.0
N3—Ni2—N4	94.64 (17)	C8—N4—C9	113.8 (4)
N3^ii^—Ni2—N4^ii^	94.64 (17)	C9—N4—Ni2	105.9 (3)
N7^ii^—Ni2—N7	180.0	C9—N4—H4	107.0
N4^ii^—Ni2—N7^ii^	89.87 (17)	C3—C2—H2A	108.1
N4^ii^—Ni2—N7	90.13 (17)	C3—C2—H2B	108.1
N4—Ni2—N7^ii^	90.13 (17)	C1—C2—C3	116.7 (4)
N4—Ni2—N7	89.87 (17)	C1—C2—H2A	108.1
N4^ii^—Ni2—N4	180.0	C1—C2—H2B	108.1
N7—C13—Ni4	176.4 (5)	H2A—C2—H2B	107.3
C11—N5—Ni1	166.1 (4)	N3—C6—H6A	109.2
H8A—C8—H8B	107.9	N3—C6—H6B	109.2
N4—C8—H8A	109.2	N3—C6—C7	111.9 (4)
N4—C8—H8B	109.2	H6A—C6—H6B	107.9
N4—C8—C7	112.1 (5)	C7—C6—H6A	109.2
C7—C8—H8A	109.2	C7—C6—H6B	109.2
C7—C8—H8B	109.2	N1^i^—C5—C4	109.3 (4)
Ni2—N3—H3	106.9	N1^i^—C5—H5A	109.8
C10^ii^—N3—Ni2	105.8 (3)	N1^i^—C5—H5B	109.8
C10^ii^—N3—H3	106.9	C4—C5—H5A	109.8
C10^ii^—N3—C6	113.3 (4)	C4—C5—H5B	109.8
C6—N3—Ni2	116.6 (4)	H5A—C5—H5B	108.3
C6—N3—H3	106.9	C10—C9—H9A	110.1
Ni1—N2—H2	107.0	C10—C9—H9B	110.1
C3—N2—Ni1	116.2 (3)	N4—C9—C10	108.2 (5)
C3—N2—H2	107.0	N4—C9—H9A	110.1
C3—N2—C4	113.7 (4)	N4—C9—H9B	110.1
C4—N2—Ni1	105.5 (3)	H9A—C9—H9B	108.4
C4—N2—H2	107.0	C8—C7—C6	117.0 (4)
N5—C11—Ni3	176.5 (5)	C8—C7—H7A	108.1
H2WA—O2W—H2WB	109.4	C8—C7—H7B	108.1
N8—C14—Ni4	178.0 (5)	C6—C7—H7A	108.1
N3^ii^—C10—H10A	109.8	C6—C7—H7B	108.1
N3^ii^—C10—H10B	109.8	H7A—C7—H7B	107.3
			
Ni1—N2—C3—C2	54.8 (5)	N2—C4—C5—N1^i^	56.3 (6)
Ni1—N2—C4—C5	−40.9 (5)	C10^ii^—N3—C6—C7	−177.7 (5)
Ni1—N1—C1—C2	−56.5 (5)	N1—C1—C2—C3	71.8 (7)
Ni2—N3—C6—C7	−54.5 (5)	C3—N2—C4—C5	−169.4 (4)
Ni2—N4—C9—C10	−40.9 (5)	C4—N2—C3—C2	177.5 (4)
C8—N4—C9—C10	−169.0 (4)	N4—C8—C7—C6	−70.7 (6)
N3^ii^—C10—C9—N4	57.3 (6)	C5^i^—N1—C1—C2	−179.1 (4)
N3—C6—C7—C8	69.7 (6)	C7—C8—N4—Ni2	55.4 (5)
N2—C3—C2—C1	−70.5 (7)	C7—C8—N4—C9	178.2 (4)

Symmetry codes: (i) −*x*+1, −*y*+1, −*z*; (ii) −*x*+1, −*y*, −*z*+1; (iii) −*x*, −*y*+2, −*z*; (iv) −*x*, −*y*+1, −*z*+1.

### Hydrogen-bond geometry (Å, º)

**Table:**

*D*—H···*A*	*D*—H	H···*A*	*D*···*A*	*D*—H···*A*
N1—H1···O1*W*^iv^	0.98	2.28	3.115 (6)	143
N2—H2···O1*W*^v^	0.98	2.10	3.020 (6)	156
N3—H3···O2*W*^iv^	0.98	2.15	3.083 (7)	159
N4—H4···O2*W*^vi^	0.98	2.26	3.080 (6)	140
O1*W*—H1*WA*···N8	0.85	2.03	2.872 (7)	173
O1*W*—H1*WB*···N6^vii^	0.85	2.27	3.112 (7)	171
O2*W*—H2*WA*···N6	0.85	2.03	2.853 (6)	164
O2*W*—H2*WB*···N8^iv^	0.85	2.30	3.149 (7)	175

Symmetry codes: (iv) −*x*, −*y*+1, −*z*+1; (v) *x*, *y*, *z*−1; (vi) *x*, *y*−1, *z*; (vii) −*x*, −*y*+2, −*z*+1.

## References

[bb1] Barefield, E. K., Wagner, F., Herlinger, A. W. & Dahl, A. R. (1976). *Inorg. Synth*. **16**, 220–224.

[bb2] Bosnich, B., Poon, C. K. & Tobe, M. C. (1965). *Inorg. Chem.* **4**, 1102–1108.

[bb3] Černák, J., Kuchár, J., Stolárová, M., Kajňaková, M., Vavra, M., Potočňák, I., Falvello, L. R. & Tomás, M. (2010). *Transition Met. Chem.* **35**, 737–744.

[bb4] Groom, C. R., Bruno, I. J., Lightfoot, M. P. & Ward, S. C. (2016). *Acta Cryst.* B**72**, 171–179. 10.1107/S2052520616003954 PMC4822653 27048719

[bb5] Jiang, X., Tao, B., Yu, X., Wang, Y. & Xia, H. (2015). *RSC Adv.* **5**, 19034–19040.

[bb6] Kaskel, S. (2016). Editor. *The Chemistry of Metal–Organic Frameworks: Synthesis, Characterization, and Applications*, 2 volumes. Weinheim: Wiley-VCH.

[bb7] Kou, H.-Z., Si, S.-F., Gao, S., Liao, D.-Z., Jiang, Z.-H., Yan, S.-P., Fan, Y.-G. & Wang, G.-L. (2002). *Eur. J. Inorg. Chem.* pp. 699–702.

[bb8] Lampeka, Ya. D. & Tsymbal, L. V. (2004). *Theor. Exp. Chem.* **40**, 345–371.

[bb9] MacGillivray, L. R. & Lukehart, C. M. (2014). Editors. *Metal–Organic Framework Materials*. Hoboken: John Wiley and Sons.

[bb10] Macrae, C. F., Sovago, I., Cottrell, S. J., Galek, P. T. A., McCabe, P., Pidcock, E., Platings, M., Shields, G. P., Stevens, J. S., Towler, M. & Wood, P. A. (2020). *J. Appl. Cryst.* **53**, 226–235. 10.1107/S1600576719014092 PMC6998782 32047413

[bb11] Melson, G. A. (1979). Editor. *Coordination Chemistry of Macrocyclic Compounds*. New York: Plenum Press.

[bb12] Ohkoshi, S., Namai, A. & Tokoro, H. (2019). *Coord. Chem. Rev.* **380**, 572–583.

[bb13] Rigaku OD (2018). *CrysAlis PRO*. Rigaku Oxford Diffraction, Yarnton, England.

[bb14] Sheldrick, G. M. (2015*a*). *Acta Cryst.* A**71**, 3–8.

[bb15] Sheldrick, G. M. (2015*b*). *Acta Cryst.* C**71**, 3–8.

[bb16] Stackhouse, C. A. & Ma, S. (2018). *Polyhedron*, **145**, 154–165.

[bb17] Suh, M. P. & Moon, H. R. (2007). *Advances in Inorganic Chemistry*, Vol. 59, edited by R. van Eldik & K. Bowman-James, pp. 39–79. San Diego: Academic Press.

[bb18] Suh, M. P., Park, H. J., Prasad, T. K. & Lim, D.-W. (2012). *Chem. Rev.* **112**, 782–835. 10.1021/cr200274s 22191516

[bb19] Tsymbal, L. V., Andriichuk, I. L., Shova, S., Trzybiński, D., Woźniak, K., Arion, V. B. & Lampeka, Ya. D. (2021). *Cryst. Growth Des.* **21**, 2355–2370.

[bb20] Westrip, S. P. (2010). *J. Appl. Cryst.* **43**, 920–925.

[bb21] Yatsimirskii, K. B. & Lampeka, Ya. D. (1985). *Physicochemistry of Metal Complexes with Macrocyclic Ligands*. Kiev: Naukova Dumka. (In Russian)

[bb22] Zhou, H.-B., Dong, W., Zhu, L.-N., Yu, L.-H., Wang, Q.-L., Liao, D.-Z., Jiang, Z.-H., Yan, S.-P. & Cheng, P. (2004). *J. Mol. Struct.* **703**, 103–106.

